# Electroacupuncture treatment partly promotes the recovery time of postoperative ileus by activating the vagus nerve but not regulating local inflammation

**DOI:** 10.1038/srep39801

**Published:** 2017-01-04

**Authors:** Jun-fan Fang, Jian-qiao Fang, Xiao-mei Shao, Jun-ying Du, Yi Liang, Wen Wang, Zhe Liu

**Affiliations:** 1 Department of Neurobiology & Acupuncture Research, the Third Clinical College, Zhejiang Chinese Medical University, Hangzhou, China

## Abstract

Postoperative ileus (POI) after abdominal surgery significantly lowers the life quality of patients and increase hospital costs. However, few treatment strategies have successfully shortened the duration of POI. Electroacupuncture (EA) is a modern way of administering acupuncture and widely used in various gastrointestinal (GI) diseases in the world. Here, we studied the effect of EA on POI and its underlying mechanisms. Intestinal manipulation resulted in significant delays of GI transit, colonic transit and gastric emptying. Surgery also up-regulated c-fos in nucleus of the solitary tract (NTS) and induced inflammation response in the small intestine. Further, operation and inhale anesthesia inhibited NTS neuron excitation duration for the whole observation time. EA administered at ST36 indeed shortened the recovery time of GI and colonic transit, and significantly increased the gastric emptying. EA also significantly activated the NTS neurons after operation. However, there was no anti-inflammation effect of EA during the whole experiment. Finally, atropine blocked the regulatory effect of EA on GI function, when it was injected after surgery, but not before surgery. Thus, the regulatory effect of EA on POI was mainly mediated by exciting NTS neurons to improve the GI tract transit function but not by activating cholinergic anti-inflammatory pathway.

Abdominal and extra-abdominal surgery can lead to impaired motility of the entire gastrointestinal (GI) tract, which is referred to as postoperative ileus(POI)^1^. Depending on the type of surgery, POI may last over 2 weeks, with symptoms including nausea, vomiting, intolerance to food, absence of defecation, longer hospital stay^2^. Therefore, POI significantly increases the hospitalization costs.

It is becoming increasingly clear that POI is associated with a localized inflammatory reaction, which is a complex orchestrated immune response to intestinal manipulation (IM)^1^,^3^. IM induced the influx of leukocytes, mainly monocytes, into the muscularis, after surgery^4^,^5^,^6^. Secretion of cytokines (IL-6, TNF-α) from monocytes and smooth muscle inhibitory substances (NO, COX-2) contribute to the delay in intestinal transit^7^,^8^. These events delay GI transit, decrease local neuromuscular function and inhibit neurogenic pathways, thereby suppressing motility along the entire gastrointestinal tract for sustained postoperative periods^9^,^10^.

Many new therapeutic strategies for POI has been introduced in the last decade, but with limited success^11^. Local inflammatory responses during POI are subject to regulation by the vagus nerve system^12^,^13^. Electrical stimulation of the vagus efferent nerve or drug stimulation of the nucleus of the solitary tract (NTS), the parasympathetic preganglionic center, reduces the inflammatory response and POI via the cholinergic anti-inflammatory pathway^14^,^15^. However, for medical ethics and safety reasons, these stimulation methods have been tried in animals or a few patient volunteers.

Electroacupuncture (EA), which is a modern way of administering acupuncture, refers to the application of a pulsating electrical current to acupuncture needles for acupoint stimulation. It has been widely used for various GI discords in China and the West^16^,^17^,^18^. EA promotes the GI function and some reports have shown therapeutic effects of EA on POI^19^,^20^. Furthermore, EA increase the motility of GI by regulating the vagus activation^21^. If EA activated vagus nerve system before surgery, it might prevent the POI by the cholinergic anti-inflammatory pathway such as previous study. Here, we focused on the regulatory effect of EA on GI motility after abdominal surgery and investigated the role of vagus nerve in EA’s regulatory effect on POI.

## Results

### GI transit

**Figure 1:**
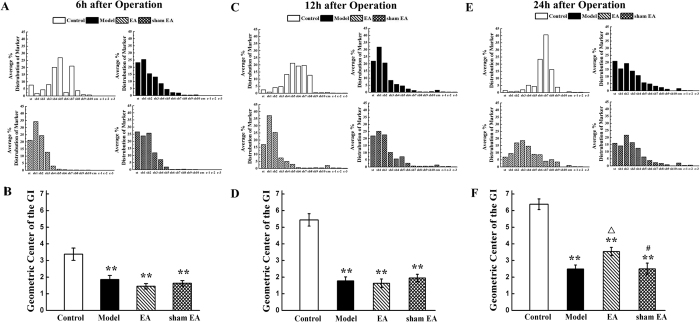
Gastrointestinal tract transit after abdomen operation. Manipulation of the rat intestine leads to a postoperative delay of intestinal transit as measured by the distribution and geometric center (GC) of fluorescent marker and EA partly recovered the gastrointestinal transit at 24 h after operation. The distribution of orally administered FITC-dextran in whole gastrointestinal tract 6 h (**A**), 12 h (**C**) and 24 h (**E**) after operation and the mean GC of fluorescent marker 6 h (**B**), 12 h (**D**) and 24 h (**F**) after surgery. n = 6–8 rats per group and data are means ± SEM. ^**^P < 0.01 versus control group, ^Δ^*P* < 0.05 versus model group, ^#^*P* < 0.05 versus EA group.

The distribution and geometric center (GC) of fluorescent markers are shown in Fig. 1. In the control group, the fluorescein isothiocyanate-labeled dextran (FITC-dextran) reached the seventh segment of the small bowel (sb-7) as reflected by a GC of 3.38 ± 0.37 at 6 h after surgical manipulation (Fig. 1A). The marker in the model group, EA group and sham EA group remained in the stomach and were distributed mainly throughout sb-3, as reflected by respective GC values of 1.86 ± 0.24, 1.45 ± 0.15, 1.63 ± 0.16 (*P* < 0.01). There was no significant difference between the model, EA and sham EA groups (*P* > 0.05) (Fig. 1B). Similarly, IM significantly delayed the GI transit of the model group, EA group and sham EA group (GC = 1.77 ± 0.24, 1.63 ± 0.26, 1.95 ± 0.22, respectively) compared with control 12 h after operation (GC = 5.44 ± 0.37, *P* < 0.01). EA had no significant therapeutic effect on gastrointestinal motility at this time point (*P* > 0.05) (Fig. 1C/D). Relative to the control group (GC = 6.38 ± 0.32), the transit distribution histograms demonstrated a significant delay of GI transit in the model, EA and sham EA groups (CG = 2.49 ± 0.24, 3.54 ± 0.25, 2.5 ± 0.34, respectively, *P* < 0.01) at 24 h after surgery. However, the fluorescent marker remaining in the stomach of the EA group was less than that of the model and sham EA groups, with a distributed to sb-6 (Fig. 1E). Although the GCs of model group, EA group and sham EA group were lower than control, the EA group’s GC was much higher than those of the model group and sham EA group (Fig. 1F, *P* < 0.05). Throughout the experiment, sham EA did not change GI transit compared with the model group (*P* > 0.05). The above data indicate that IM caused a clinically relevant POI and EA treatment partly mitigated the delay of GI transit up to 24 h after surgery.

### Colonic transit

**Figure 2:**
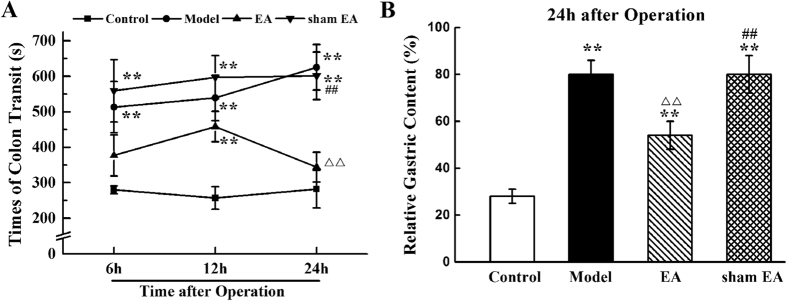
Colonic transit and gastric emptying after abdomen operation. Surgery manipulation of the rat small intestine produced a pronounced increase of colonic transit time of a 5-mm stainless steel ball and a significantly increase of gastric retention. EA stimulation at ST36 reversed them. The mean colonic transit time of a stainless steel ball was calculated 6 h, 12 h and 24 h after surgery (**A**) and the average gastric retention 24 h after operation (**B**). n = 6–8 rats per group and data are means ± SEM. ^**^P < 0.01 versus control group, ^ΔΔ^*P* < 0.01 versus model group, ^##^*P* < 0.01 versus EA group.

The mean colonic transit times in all experimental groups at each time point are shown in Fig. 2A. The repeated-measures ANOVA with between-subject factors revealed differences over time (*P* < 0.01) and between groups (*P* < 0.01). There were significant interactive effects between time points and groups (*P* < 0.01). Post-hoc LSD tests indicated that IM caused a delay of colonic transit in rats (*P* < 0.01). A significant difference of colonic transit time was observed in the EA group compared with the model group (*P* < 0.01). However, sham EA stimulation did not change the colonic transit time of rats compared with the model group (*P* > 0.05).

Comparing colonic transit time between groups at each time point, significant differences occurred among the control, model, EA and sham EA groups from 6 h to 24 h after IM (*P* < 0.01). Compared with the control group, IM significantly delayed the colonic transit of the model and sham EA groups during the observation time (*P* < 0.01). However, IM only significantly increased the EA group’s colonic transit time only 12 h after surgery (*P* < 0.01). Following the EA treatment, the colonic transit time was significantly decreased compared the model and sham EA groups 6 h after surgery (*P* < 0.05). Furthermore, 24 h after IM, the colonic transit function of the EA group recovered to normal level (*P* > 0.05).

### Gastric emptying

The mean gastric emptying levels in all experimental groups at 24 h after operation are shown in Fig. 2B. Manipulation of the small intestine initiated a significant increase in gastric retention 24 h after surgery compared with control (*P* < 0.01). Compared with the model group, EA treatment significantly decreased the gastric retention 24 h after IM (*P* < 0.01), but sham EA stimulation did not change the gastric function (*P* > 0.05). However, there was still a significant difference between the EA group and the control group 24 h after IM (*P* < 0.01).

### Expression of c-fos in NTS

**Figure 3:**
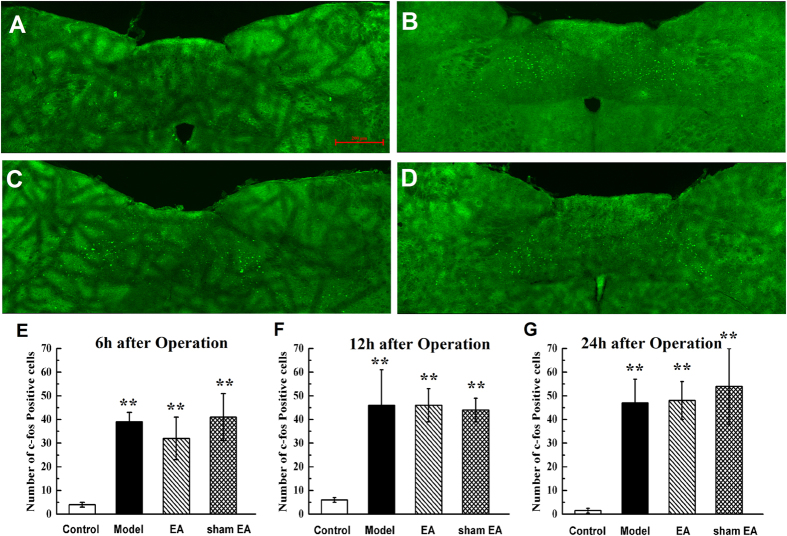
c-fos expression in nucleus of solitary tract after abdomen operation. Manipulation of the gastrointestinal tract promoted the c-fos expression level of bilateral nucleus of solitary tract (NTS), however, EA simulation produced little effect on these change. Immunofluorescence staining of c-fos expression at bilateral NTS from rats that underwent a laparotomy alone (**A**), a laparotomy with intestinal manipulation (**B**), a intestinal manipulation with EA stimulation (**C**) and intestinal manipulation with sham EA stimulation (**D**) 24 h after operation. The mean number of positive reaction cells of c-fos from NTS 6 h (**E**), 12 h (**F**) and 24 h (**G**) after operation. Scale bar represent 200 μm. n = 6–8 rats per group and data are means ± SEM. ^**^P < 0.01 versus control group.

To obtain more insights into the central mechanism by which EA regulates POI, immunofluorescence analysis of the brain for c-fos was performed. The expression of c-fos in the NTS at 6 h, 12 h and 24 h after IM is shown in Fig. 3. High expression of c-fos in the NTS were found at 24 h after surgery in all rats that underwent abdominal operation (Fig. 3A–D). Quantitative c-fos analysis 6 h, 12 h and 24 h after IM showed great number of c-fos-positive neurons in the model group, EA group and sham EA group compared with the control group (*P* < 0.01, Fig. 3E–G). EA and sham EA stimulation did not produce any regulatory effect on c-fos expression at 6 h, 12 h and 24 h after surgery compared with the model group (*P* > 0.05, Fig. 3E–G).

### NTS exciting in POI rats

**Figure 4:**
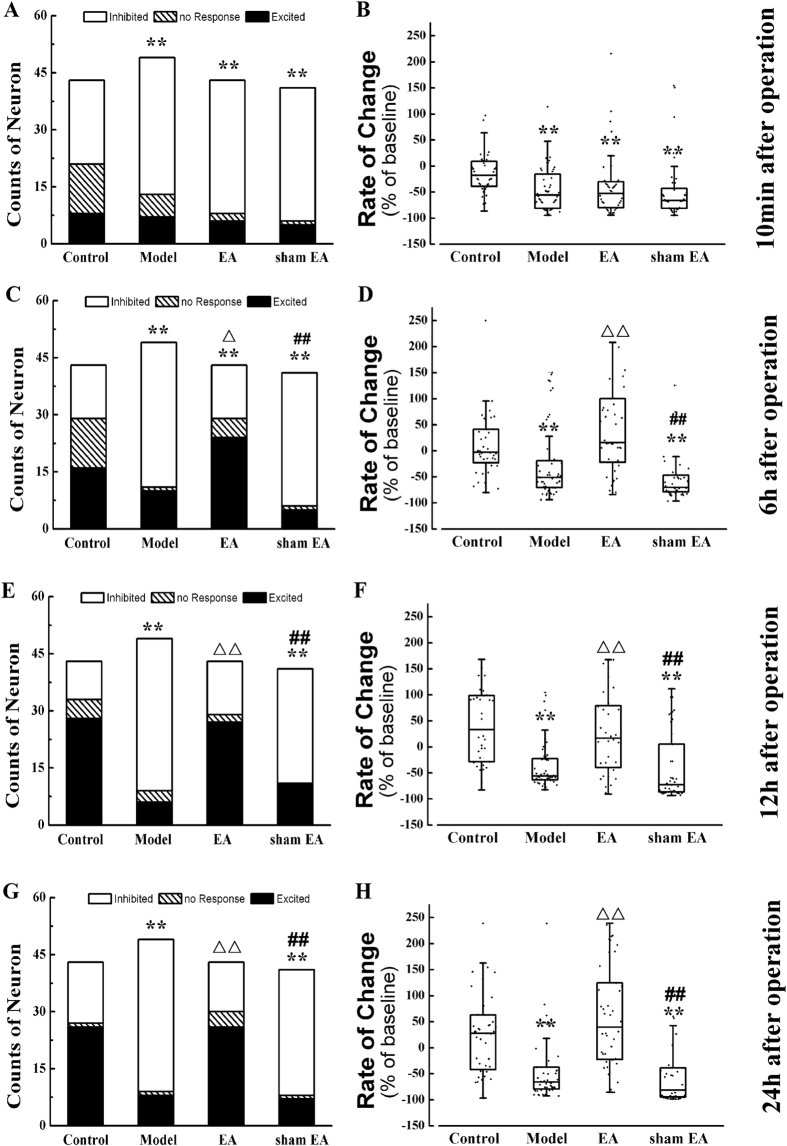
Nucleus of solitary tract neurons excitation after abdomen operation. Manipulation of the gastrointestinal tract and the inhaled anesthetized suppressed the NTS neurons excitation till 24 h after operation and EA excited the NTS. The distribution of three types neuron in NTS of four group immediately (**A**), 6 h (**C**), 12 h (**E**) and 24 h (**G**) after operation and the rate of change of spike frequency of NTS neurons 10 min (**B**), 6 h (**D**), 12 h (**F**) and 24 h (**H**). Results of three type neurons’ number are presented as count data and results of the rate of change of spike frequency are mean, quartile and standard deviation. ^**^P < 0.01 versus control group, ^ΔΔ^*P* < 0.01 versus model group, ^##^*P* < 0.01 versus EA group.

We next investigated the effect of EA on the NTS of POI rats by *in vivo* electrophysiology. One hundred sixty-six cingulate neurons from 20 rats were recorded. Forty-three were from the control group, 49 were from the model group, 43 were from the EA group and 41 from the sham EA group. The number of three type neurons (excited, inhibited, and no response) and the rate of change of spike frequency are shown in Fig. 4.

More than 50% of the NTS neurons of rats in the control group were inhibited immediately after the surgery (Fig. 4A). These results indicate that only isoflurane, without small bowel manipulation, down-regulated the NTS neuron excitability, which was consistent with the inhibitory effect of isoflurane on GI. However, the effect of isoflurane faded by 6 h after the surgery, and the numbers of the three types NTS neurons in the control group tended to equilibrium (Fig. 4C). As with GI function, small bowel manipulation significantly inhibited the NTS neuronal excitability and decreased the spike frequency when compared with the control group during the whole observation time (*P* < 0.01, Fig. 4A–H). EA and sham EA stimulation did not produce any significantly effect on NTS neuronal excitability or spike frequency compared with the model group immediately after operation (*P* > 0.05, Fig. 4A,B). However, EA stimulation significantly up-regulated the NTS neuronal excitability and increased the spike frequency compared with the control and model groups at 6 h after operation (*P* < 0.05, Fig. 4C,D). Similarly, from 12 h to 24 h, EA significantly excited the NTS neurons and increased the spike firing frequency compared with the model group (*P* < 0.01, Fig. 4E,H). However, no distinct difference between the EA and control groups was observed at the above time points (*P* > 0.05, Fig. 4E,H).

### NTS excitation in normal rats

**Figure 5:**
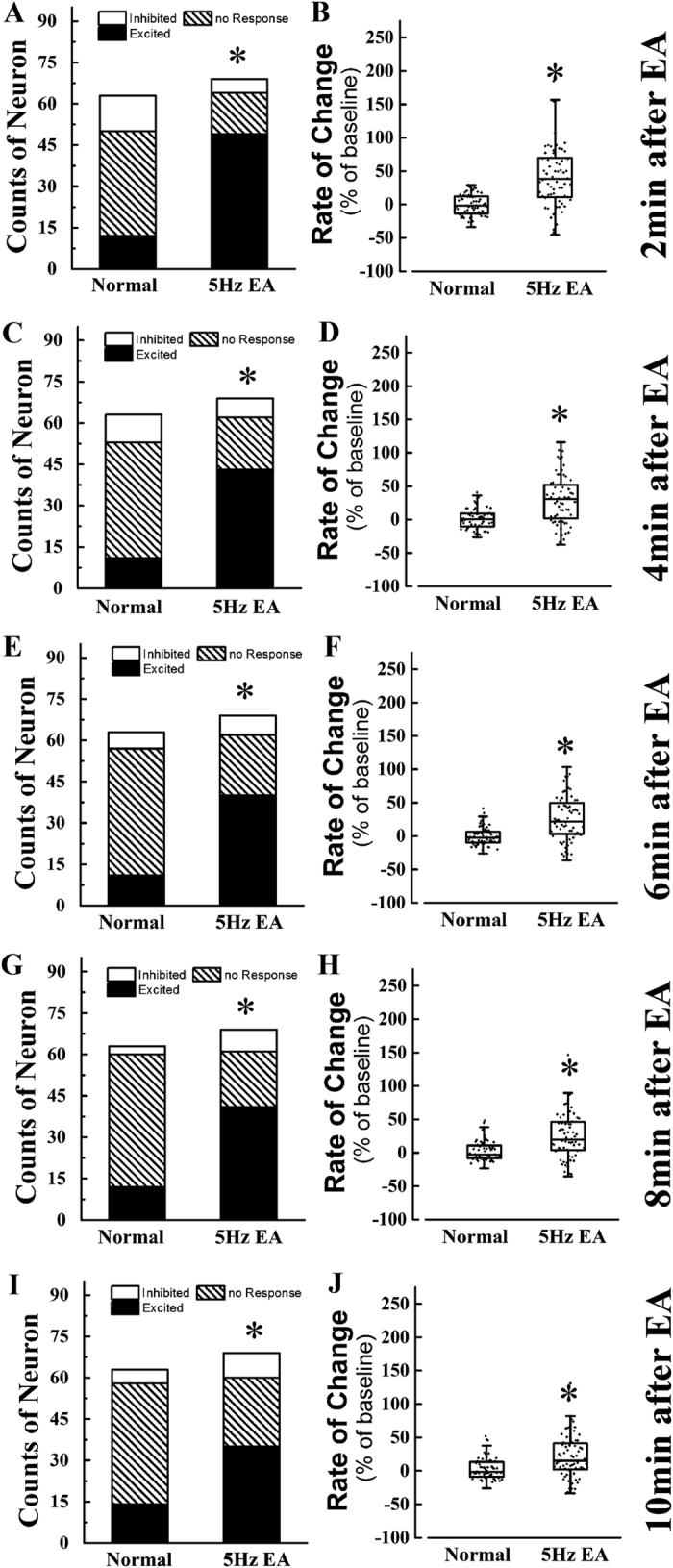
EA excitatory effect on nucleus of solitary tract neurons on normal rats. EA stimulation excited the NTS neurons when administrated to normal rats. The distribution of three types neuron in NTS of four group 2 min (**A**), 4 min (**C**), 6 min (**E**), 8 min (**G**) and 10 min (**I**) after EA stimulation and the rate of change of spike frequency of NTS neurons 2 min (**B**), 4 min (**D**), 6 min (**F**), 8 min (**H**) and 10 min (**J**). Results of three type neurons’ number are presented as count data and results of rate of change of spike frequency are mean, quartile and standard deviation. ^*^P < 0.05 versus normal group.

To investigate the effect of 5 Hz EA stimulation at ST36 on NTS neurons before surgery, we studied EA’s effect on normal rats. One hundred thirty-two cingulate neurons from 10 rats were recorded. Sixty-three were from the control group, and 69 were from the EA group. The numbers of the three types of neurons and the rate of change of spike frequency are shown in Fig. 5. Throughout the observation time, more than 50% of the neurons showed no response in the normal group (Fig. 5A,C,E,G,I), which indicated that the NTS neuronal excitability tend to stabilize without stimulation (Fig. 5B,D,F,H,J). After 30 min of 5 Hz EA stimulation, more than 50% of the neurons in the NTS were excited (Fig. 5A,C,E,G,I), and the NTS neuronal excitability and spike frequency were significantly increased (*P* < 0.05, Fig. 5B,D,F,H,J). These findings indicate that 5 Hz EA simulation at ST 36 produced a persistent excitation on NTS neurons that lasted at least 10 min.

### Leukocyte infiltration into the intestinal wall

**Figure 6:**
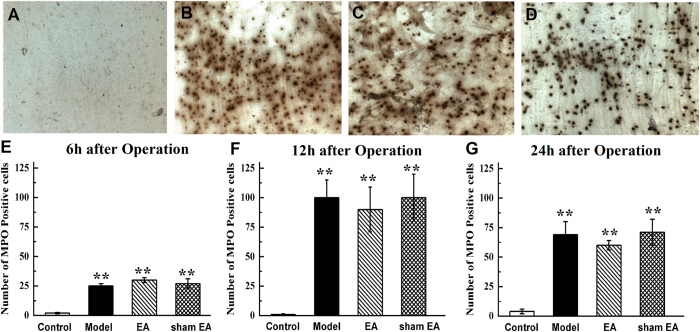
Leukocyte infiltration in intestinal wall after abdomen operation. Manipulation of the gastrointestinal tract produced a pronounced leukocyte infiltration in intestinal wall from 6 h to 24 h after surgery and EA stimulation had little effect on infiltration. MPO staining of muscle whole mounts from rats that underwent a laparotomy alone (**A**), a laparotomy with intestinal manipulation (**B**), an intestinal manipulation with EA stimulation (**C**) and intestinal manipulation with sham EA stimulation (**D**) 24 h after operation. The mean number of MPO-positive cells in small intestine 6 h (**E**), 12 h (**F**) and 24 h (**G**) after operation. Scale bar represent 20 μm. n = 6–8 rats per group and data are means ± SEM. ^**^P < 0.01 versus control group.

Myeloperoxidase (MPO) staining at each time point is shown in Fig. 6. IM induced a significant increase in the number of MPO-positive cells in the intestinal wall 6 h after IM compared with laparotomy alone (*P* < 0.01, Fig. 6E). This leukocyte recruitment was not regulated by EA or sham EA stimulation compared with the model group (*P* > 0.05, Fig. 6E). Furthermore, the influx of inflammatory cells in the model group was even more pronounced at 12 h (Fig. 6A–D) and 24 h after surgery (*P* < 0.01, Fig. 6F,G). Most importantly, no anti-inflammatory effect of EA treatment was observed during the experiment compared with the model and sham EA groups.

### Expression of IL-1β and TNF-α mRNA

**Figure 7:**
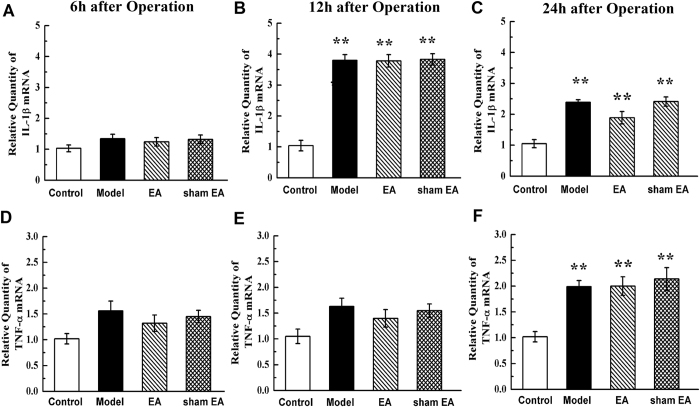
IL-1β and TNF-α mRNA expression in small intestinal after abdomen operation. Manipulation of the gastrointestinal tract produced a pronounced IL-1β and TNF-α mRNA expression in small intestinal from 6 h to 24 h after surgery and EA stimulation had little regulation. IL-1β mRNA relative expression level in intestinal wall of four group 6 h (**A**), 12 h (**B**) and 24 h (**C**) after operation. TNF-αmRNA relative expression level in intestinal wall of four group 6 h (**D**), 12 h (**E**) and 24 h (**F**) after operation. n = 6–8 rats per group and data are means ± SEM. ^**^P < 0.01 versus control group.

Along with the leukocyte recruitment to the intestinal wall from 6 h to 24 h after surgery, we chose this period to test the effect of EA on some proinflammatory gene expression. There were no differences in the expression of IL-1β and TNF-α mRNA expression at 6 h after surgery (*P* > 0.05, Fig. 7A,D). However, intestinal manipulation up-regulated the IL-1β mRNA expression from 12 h to 24 h after surgery compared with control (*P* < 0.01, Fig. 7B,C). IM also increased TNF-α mRNA 24 h after surgery (*P* < 0.01, Fig. 7F). The expression of IL-1β and TNF-α in the EA group showed no significant difference from either the model group or the sham EA group for the full observation time (*P* > 0.05, Fig. 7A–F).

### The atropine anti-EA regulation effect

**Figure 8:**
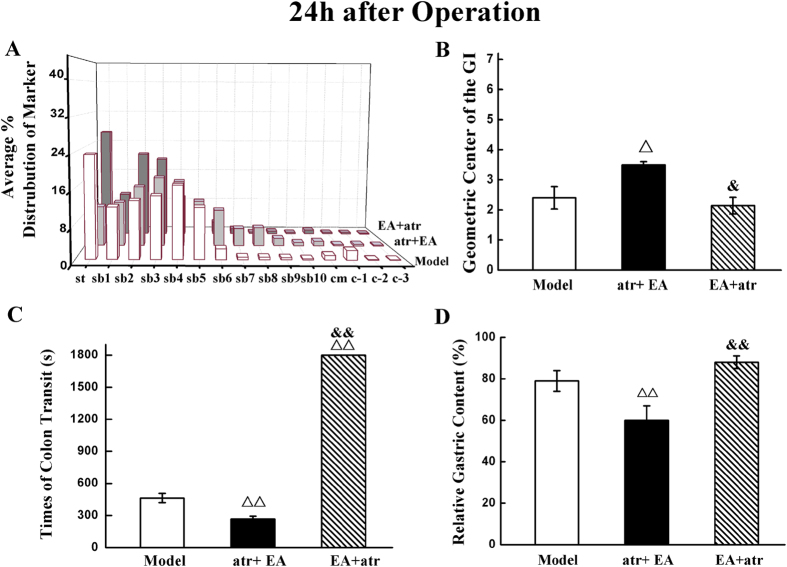
Atropine inhibit EA’s effect. EA’s regulatory effect on gastrointestinal transit, colonial transit time and gastro emptying was inhibited by atropine when it was administrated after operation, but not before. The distribution of orally administered FITC-dextran in whole gastrointestinal tract (**A**) and the mean GC of fluorescent marker (**B**) 24 h after surgery. The mean colonic transit time (**C**) and the average gastric retention 24 h after operation (**D**). n = 6–8 rats per group and data are means ± SEM. ^Δ^*P* < 0.05, ^ΔΔ^*P* < 0.01 versus model group, ^&^*P* < 0.05, ^&&^*P* < 0.01 versus atr + EA group.

The above results indicated that EA may promote GI transit function through the vagus nerve, so we injected atropine intraperitoneally before EA administration. As shown in Fig. 8A,B, the distribution of marker in the atropine plus EA group was different from the model group and EA plus atropine group, and the GC of the atropine plus EA group was much higher than those of model group and EA plus atropine group (*P* < 0.05). There was no significant difference between the model and EA plus atropine group (*P* > 0.05). Gastric emptying and colonic transit testing showed similar results (Fig. 8C,D). EA simulation significantly decreased the relative gastric content and colonic transit time of POI rats when the atropine was injected before operation (*P* < 0.01). However, atropine given after surgery inhibited EA enhancing effect on gastric emptying and colonic transit (*P* > 0.05). Furthermore, administration atropine significantly delayed the colon transit of rats (*P* < 0.01). We found the stainless steel ball in the colon when the rats were sacrificed.

## Discussion

POI has been characterized by GI dysfunction and a series of clinical symptom, such as abdominal pain and distension, oral diet intolerance, and passage of flatus and stool delay^22^. Because of its adverse clinical consequences, POI is one of the most important contributors to longer hospital stays and higher costs^23^. A way to promote recovery of GI function after abdominal surgery has been desired for decades, but the results have been disappointing^11^,^24^. In the present study, we demonstrated that EA stimulated on Zusanli (ST36) improved post-operative intestinal transit, increased the post-operative gastric emptying and shortened the time of post-operative cacation. EA promoted NTS excitation after abdominal surgery but did not reduce the inflammatory cell influx or the local inflammation of small intestine in response to IM. Our results are line with earlier studies suggesting that EA regulated the GI function via vagus nerve system^25^. However, our results contrasted with findings that activating the vagus before surgery reduce the inflammation of the small intestine.

It is generally believed that three factors suppress GI motility after abdominal surgery^26^. They are adrenergic and noradrenergic inhibitory neuronal pathways that activated by the nociceptive stimuli during surgery^1^,^27^, anesthetics and opioid analgesics used during and after the surgery^28^,^29^,^30^, and the local inflammatory response to intestinal handling during the surgery^5^. Anesthetics were once thought to be the main factor leading to POI. Now, the local inflammation of the intestine, induced by IM, is considered the main mechanism underlying POI and the most viable therapy target^31^. The relationship between intestinal inflammation and POI was first reported at the end of the 20^th^ century^3^,^32^,^33^. Then, Kalff first demonstrated the correlation between inflammation and POI in the human body at 2003^23^. Manipulation of the intestine during the abdominal surgery, including laparoscopic surgery^34^, activates the resident innate immune cells located within the muscularis externa, which initiates the circulating leukocytes invading the small intestinal wall^1^,^26^,^35^,^36^. The infiltration of leukocytes is not restricted to the location of handling but fast spreads to all areas of the small intestine^37^. The mechanisms of the inhibition of GI motility by this local inflammation are not clear yet. The leukocytes and resident innate immune cells released various cytokines and chemokines^4^,^36^,^38^, which not only compromised the contractile activity but also excite the inhibitory neural pathways^5^,^6^ and inhibit the vagus nerve system affecting the entire gastrointestinal tract^39^. Here, the transit function of the whole GI tract was significantly suppressed from 6 h to 24 h after surgery, with leukocytes infiltrated into the small intestinal muscular layer. These results are consistent with previous studies supporting the relationship between local inflammatory response and POI. Interestingly, the small intestinal motility function of control rats gradually recovered from 6 h to 12 h after operation, which was in line with the previous results showing that anesthetics are involved in the early POI. Thus, we believe that the delay of GI transition in the model rats was induced by both anesthetics and inflammatory response up to 6 h after surgery and that the persistent local inflammation of the small intestine induced the POI from 12 h to 24 h after surgery.

Although the inflammation of the intestine was thought to underlie POI, traditional inflammatory agents failed to cure POI^40^,^41^. The vagus nerve system regulates the systemic and local inflammatory response^42^,^43^, which is currently referred to as the cholinergic anti-inflammatory pathway. The anti-inflammatory effect of the vagus is mediated by acetylcholine, which interacting with α-7 submit nicotinic receptors located on macrophages and inhibit the cytokine production^44^,^45^. For the past decade, the cholinergic anti-inflammatory pathway has been used to treat sepsis, inflammatory bowel and pancreatitis^46^,^47^,^48^, and recently was introduced for POI treatment^49^,^50^. Vagus nerve stimulation can prevent the inflammation response to surgery and alleviated prolonged POI^15^,^51^. However, the methods of stimulation of the vagus nerve, either electro-stimulation on efferent vegus fiber or intracerebroventricular injection (i.c.v.), are difficult to apply clinically.

EA, a traditional Chinese medical treatment, is applied to various GI disease without adverse effect. Here, we introduced it for POI treatment because a growing body evidence indicates that EA regulates the GI function by exciting vagus nerve system and might produce anti-inflammatory effect^52^,^53^. If EA could activate vagus nerve system before surgery, it might prevent POI via the cholinergic anti-inflammatory pathway such as previous study. Our results show that EA indeed recovered small intestinal motility function at 24 h after surgery and significantly promoted the colon transit function and gastric emptying. All of these results indicate that EA shortened the recovery time of the stomach, small intestine and colon, as supported by clinical reports^54^. To learn the mechanisms underlying the treatment effect of EA on POI and further enhance it, we detected the excitation of the NTS, the central nucleus of the vagus reflex, by electrophysiology and c-fos expression to investigate the vaugs nerve excitation.

The NTS may be activated after abdominal surgery^31^, and the brain may sense peripheral inflammation partly through vagal afferents and produce an anti-inflammatory response via vagal efferent fibers^55^. The intestinal inflammatory response to IM and the NTS activation during POI has been demonstrated^15^,^56^. Thus, the vagus nerve system may be activated by the inflammation in small intestine to reduce the inflammatory action via the cholinergic anti-inflammatory pathway. In our results, c-fos expression in the NTS was significantly increased after operation at all surgery rats. However, the results of electrophysiology were different from the immunofluorescence results in this study. The spike frequency and population of excited NTS neurons were significantly suppressed after surgery. In particularly, the exciting of NTS neurons in control rats was suppressed at immediately and 6 h after operation, which was line with the generally belief that anesthesia inhibits central nerve system^57^ and the result that GI transition was weakened at 6 h after operation in the control group. In contrast, a low exciting of NTS neurons from 6 h to 24 h after operation was consistent with the previous result that abdominal surgery excited the NANC pathway inhibit vagus nerve^39^. Furthermore, the control rats’ NTS exciting was strongly excited from 6 h to 24 h, which may be induced by hunger. However, there were little c-fos expression in NTS in control rats. We think that NTS, as a parasympathetic center of the vago-vagal reflex, detected the inflammatory response at the intestinal wall but some factors, such as anesthesia and inhibitory neurons, might block its excitation. The relationship between c-fos expression and the neuronal exciting electric signaling will be studied in our further research.

When investigated the EA’s effect on NTS, we observed that EA stimulation did not regulate the c-fos expression in the NTS at any time point. However, EA significantly increased the NTS exciting from 6 h to 24 h after surgery. Unfortunately, we did not observe any effect of EA on local inflammation. The leukocyte invasion into the intestinal wall from 6 h to 24 h after surgery and peaked at 12 h after operation. Although EA activated the NTS at 6 h, 12 h and 24 h after operation, it did not decrease the level of leukocyte infiltration. Activation of the resident macrophages is the first step in leukocyte infiltration of the small intestine^26^,^58^. Hence, vagus stimulation prevented the local inflammatory response via inhibition of macrophage activation by the cholinergic anti-inflammatory pathway. Our results show the excitatory effect of EA on NTS in normal rats, which was consistent with previous studies^25^,^52^. However, the NTS excitation effect of EA was detected at 6 h, not immediately after surgery. These results indicate that the effect of EA, administered before and during the surgery on NTS was interfered with some factors. EA is generally considered to excite the NTS via somatic afferents, then activated the vagus efferent fibers, inducing acetylcholine release. So EA’s effect may have been disturbed by the anesthetic effect of isoflurane, whether on NTS or the peripheral nerve system. However, the cholinergic anti-inflammatory pathway mainly affects macrophages, not leukocyte. When leukocyte had invaded the intestinal wall, EA administered at 6 h after operation failed to prevent inflammation. All the above are the reason why we think EA indeed excited the NTS but failed to prevent inflammation. Because EA failed to regulate the inflammatory response, we did not further investigate the effect of EA on muscle inhibitory substances.

Finally, we combine EA treatment with an intraperitoneal injection of atropine before or after abdominal surgery, to investigate the main mechanism underlying EA’s effect on POI. The results indicate that suppressing the vagus nerve system before operation, which also blocked the cholinergic anti-inflammatory pathway, did not affect the regulatory effect of EA on POI. In contrast, atropine significantly blocked the regulatory effect of EA on POI when injected after operation. We believed that the therapeutic effect of EA on POI mainly depended on the NTS-promoting effect in the GI tract, which was induced by EA administered after operation, but not the anti-inflammation effect of vagus excitation that might be produce by preoperative EA.

We showed that EA reduced the recovery time of POI after abdominal surgery. This effect is mainly mediated by the exciting of the NTS to improve GI transit function. However, we failed to prevent the local inflammation of the small intestine by the vague excitation effect of EA treatment. Further exploration of EA administration methods that activate the cholinergic anti-inflammatory pathway after abdominal operation may improve the treatment effect of EA on POI and are the subject of ongoing investigation.

## Methods and Materials

### Animals and groups

All rats were obtained from the animal experiment center attached to Zhejiang Chinese Medical University. Rats used in electrophysiological experiments weighted 280–300 g, and others were 200–220 g. They were housed with an artificial 12-h light-dark cycle at a controlled temperature (23 ± 1 °C) and relative humidity (70 ± 10%). Distilled water and food were available ad libitum. Rats were fasted from 12 h before surgery to 24 h after surgery with water available ad libitum. All animal care, surgery, and handling procedures were approved by the animal experiment center attached to Zhejiang Chinese Medical University and performed strictly in accordance with the National Institutions of Health Guide for the Care and Use of Laboratory Animals.

### Surgical procedures

POI was induced by a standardized small bowel manipulation procedure^7^. Rats were anesthetized with inhaled isoflurane, and the abdomen was opened by midline laparotomy. The small intestine was carefully exteriorized and then gently manipulated along its entire length using moistened sterile cotton applicators. Finally, the bowel was repositioned in the abdominal cavity, and the incision was closed by a continuous 2-layer suture. The rats were allowed to recover in the same cage.

### Electrode implantation surgery

Rats were deeply anesthetized with urethane (1 g/kg, i.p.) and transferred to a stereotaxic instrument. Craniotomy was made for microelectrode array implantation in one side of the brain. According to the atlas of Paxinos and Watson (Edition VI), the NTS was located as follows: 12.8 mm posterior to bregma, 0.8 mm lateral to the midline, and 5.8 mm ventral to the skull surface. An array of eight stainless steel Teflon-insulated microwires (50μm thick) were slowly lowered into the NTS. The microelectrode arrays were secured onto the cranium with stainless steel skull screws and dental cement. Rats were administered penicillin (20,000 U, i.m.) and allowed 7 days to recover.

### EA and sham EA stimulation

During the whole EA stimulation, rats were loosely immobilized by an assistant’s hands, except during the surgical process. Four stainless steel acupuncture needles 0.25 mm in diameter were inserted at a depth of 5 mm into the bilateral “Zusanli” (ST36, between the tibia and fibula 5 mm below the knee) acupoints and reference points (1 cm below ST 36). The two ipsilateral needles were connected to the output terminals of the HANS Acupuncture point Nerve Stimulator (LH-202H, Huawei Co., Ltd., Beijing, China). Electro-stimulation was delivered with constant parameters, constant square wave current output at 5 Hz (pulse width: 0.2 ms), and intensities ranging from 1 to 2 mA (each intensity for 15 min, totaling 30 min). The sham EA group received the same subcutaneous needle insertion (2 mm in depth) into ST36 and reference points, and the needles were linked to the electrodes but without electrical stimulation. To eliminate the stress effect, rats in the model and sham EA groups were loosely immobilized by an assistant’s hands similar to the EA group.

### Gastrointestinal transit

To determine the effects of IM and EA on gut motility, GI transit was measured at 6 h, 12 h and 24 h after IM as previously described^7^. Two hundred microliters of a liquid non-absorbable FITC-dextran (70,000 Da, Sigma-Aldrich, USA) was administered via oral gavage. Forty minutes later, rats were sacrificed, and the entire gastrointestinal tract from stomach to distal colon was collected. The contents of the stomach, small bowel (equal divided into 10 segments), cecum, and colon (equal divided into 3 segments) were flushed and collected. Finally, the fluorescent signal in each bowel segment was quantified in duplicate (excitation wavelength: 485 nm, emission wavelength: 528 nm) by a microplate reader (Spectra Max M4, Molecular Devices, USA). For statistical analysis, a geometric center (GC) was calculated to represent the GI function: Σ (% of total fluorescent signal in each segment × the segment number)/100.

### Colonic transit

Colonic function was investigated *in vivo* 6 h, 12 h and 24 h after IM by measuring the colon transit time of a stainless steel ball. A 5 mm diameter stainless steel ball was carefully inserted 4.5 cm into the colon with a polished metal rod via the anus. The time from insertion until excretion of the ball was considered the colonic transit time.

### Gastric emptying

The gastric emptying was measured at 24 h after IM by a semi-liquid, non-caloric test meal (2 mL of 1.5% methylcellulose solution containing 0.1% phenol red). Animals were sacrificed 40 min after oral gavage and the stomach was immediately removed with gastro-esophageal and gastroduodenal clamping. The stomach was rinsed with phosphate-buffered saline (PBS, pH 7.4) and dissected in 18 mL normal saline (NS). Twenty milliliters of 0.5 M NaOH was added to the NS, and the contents of the stomach, containing phenol red solution, were mixed. Five milliliters of supernatant was collected, and 0.5 mL of 20% trichloroacetic acid was added to precipitate proteins. The samples were centrifuged at 3600 rpm for 10 min, and the absorbance of samples at 560 nm wavelength was measure with a spectrophotometer (Spectra Max M4, Molecular Devices, USA). A control sample was prepared as follow: 2 mL 1.5% methylcellulose solution containing 0.1% phenol red, 18 mL NS, 20 mL 0.5 M NaOH and 4 mL 20% trichloroacetic acid. The absorbance of the control sample at 560 nm wavelength was also measured. The gastric emptying was estimated from the following formulation: (1 − absorbance of residual phenol red in stomach absorbance of control phenol red) × 100%.

### *In vivo* electrophysiological recording

The neuronal activities were investigated before and after the EA stimulation. During the recording session, rats were moving freely. The neuronal activities were detected by the microwires and passed from the headstage to a preamplifier. Single activities were recorded using a 128-channel data acquisition system (Cerebus, Blackrock Microsystems, USA). The neural signals were analog-filtered by the amplifier at cutoff frequencies of 0.3 Hz and 7.5 kHz and digitized at a 16-bit resolution at 30,000 Hz using a Cerebus Neural Signal Processor. The digitized signals from each microwire were amplified and digitized bandpass filtered from 250 Hz to 5000 Hz. Finally, all the signals were saved into a data file for off-line analysis. Spike activities were extracted from the digitized recordings, and individual units were isolated offline using Plexon Offline Sorter. A signal-unit was defined by homogenous waveforms quantified by sets of waveform parameters clustered in a multidimensional parameter space. The waveform parameters were auto-set by the K-means method, which was built into the software.

Ten minute *in vivo* electrophysiological recordings were performed on each rat before EA was administered, to characterize the neural activity in the NTS and calculate the average spike firing frequency (baseline). NTS neurons were grouped into three different types according to their changes: excited, inhibited and no response. For excited neurons, the firing frequency of spikes increased more than 15% over baseline after the application of EA stimulation. For inhibited neurons, the frequency of spikes decreased more than 15% from baseline. All three types of neurons were counted, and their distributions were calculated and compared to analyze the NTS neurons’ excitability.

### Immunofluorescence

Six, twelve or twenty-four hours after IM, rats were sacrificed by perfusion with NS through the ascending aorta, followed by injection of 4% paraformaldehyde. The brainstem was quickly collected and post-fixed for 24 h with the fixing solution at 4 °C, and cryo-protected by immersion in 30% sucrose. Coronal brainstem sections (30 μm thick) were collected. After rinsing in 0.1 M PBS (pH 7.4), sections were blocked with 5% normal donkey serum with 0.1% Triton X-100 for 1 h at 37 °C and incubated overnight at 4 °C with rabbit anti-c-fos primary antibodies(1:3000, abcam, USA). The sections were then incubated for 1 h with Alexa Fluor 488-labeled secondary antibody (1:1000, abcam, USA). Images were captured from NTS at 10 × magnification using a NIKON A1R laser confocal microscopy. At least 5 nonadjacent sections were used to for counting the c-fos expression in the NTS by an observer blind to the treatment.

### mRNA expression

The mRNA expression was investigated 6 h, 12 h and 24 h after IM. After harvesting the rat’s intestine, the attached mesentery and muscularis externa was removed using moist cotton applicators. The muscularis tissue samples were then stored at −80 °C. We performed real-time reverse transcriptase-polymerase chain reaction (qPCR) for IL-1β and TNF-αin the intestinal muscularis (small bowel) at 6 h, 12 h and 24 h after IM (n = 6 per group). Total RNA was extracted using Trizol Reagent (Invitrogen, France) by the guanidium thiocyanate method as previously described^55^. RNA was quantified by spectrophotometry. cDNA was synthesized using the iScriptTM cDNA Synthesis kit (Bio-Rad, USA). Relative mRNA levels were quantified using SsoFast EvaGreen supermix (Bio-Rad, USA) and the CFX96™ real-time PCR detection system (Bio-Rad, USA). The comparative cycle threshold Ct method was used for relative quantification of gene expression. The amount of IL-1β and TNF-α mRNAs, normalized to GAPDH and relative to a calibrator, was calculated by the 2^−ΔΔCt^ method, with Ct indicating the cycle number at which the fluorescence signal of the PCR product crossed an arbitrary threshold set within the exponential phase of the PCR, and ΔΔCt = [(Cttarget (unknown sample) − Ctend. control (unknown sample))] − [(Cttarget (calibrator sample) − Ctend. control (calibrator sample))]. The sequences of the primers were as follows:

GAPDH:sense primer: 5′-TGCTGAGTATGTCGTGGAG-3′,

anti-sense primer: 5′-GTCTTCTGAGTGGCAGTGAT-3′;

IL-1β: sense primer: 5′-GGGATGATGACGACCTGC-3′,

anti-sense primer: 5′-GAGAATACCACTTGTTGGCTTA-3′;

TNF-α: sense primer: 5′-CTGGCCAATGGCATGGATCTCAAA-3′,

anti-sense primer: 5′-ATCCTTGTCCCTTGAAGAGAACCT-3′.

### Staining for leukocyte infiltration

After harvesting the rat’s intestine, small bowel segments were cut open and rinsed in ice-cold modified Krebs buffer as previously described. To identify MPO-positive cell, the sample were fixed with 100% ethanol for 10 min. Then, segments were transferred to ice-cold modified Krebs buffer and pinned flat. Mucosa and submucosa were removed, and the remaining muscularis externa were stained for polymorphonuclear neutrophils with Hanker Yates regent (Sigma-Aldrich, USA) for 10 min. For quantification, the myeloperoxidase (MPO)-positive cells in 10 randomly chosen fields were counted by an observer blind to the treatment.

### Experimental design

Two sets of experiments were conducted to investigate the effect and mechanisms of EA on relation POI. (I) EA alleviates POI partly via activating the vagus nerve; (II) EA plus atropine intraperitoneal injection.

In experiment I, 192 rats were randomly divided into four groups: (a) control group: only opening the abdomen without IM (n = 48); (b) model group: underwent IM operation (n = 48); (c) EA group: underwent IM operation and EA treatment (n = 48); and (d) sham EA group: underwent IM operation and sham EA treatment (n = 48). First, at 6 h, 12 h and 24 h after operation, 6 rats of each group were sacrificed to detect the gastrointestinal transit. Then, 6 rats of each group were sacrificed to detect the colonic transit at 6 h, 12 h and 24 h after operation and to detect the gastric emptying at 24 h after operation. Finally, 5 rats were used in electrophysiological recording. In experiment I, EA and sham EA stimulation were administered during the entire perioperative period. EA and sham EA were administered 48 h, 24 h and 0.5 h before surgery. Then, EA and sham EA were administered during the whole surgical process (30 min). Finally, EA and sham EA were administered 6 h, 12 h and 48 h after IM. For distinguishing the rats after abdomen surgery, rats received the electrode implantation surgery and before surgery called normal rats. In experiment II, 18 rats that underwent the IM operation were randomly divided into three groups: (a) model group: only underwent IM operation (n = 6); (b) EA plus atropine group: intraperitoneal injection atropine preoperational (n = 6) and (c) EA plus atropine group: intraperitoneal injection atropine after operation (n = 6). EA stimulation was administered as in experiment I. Atropine was injected at 48 h, 24 h and 0.5 h before surgery, or 6 h, 12 h and 24 h after surgery, before EA stimulation.

### Statistical analyses

The counts of the three types of neurons are presented as count data. Spike frequency is presented as the mean, quartile and standard deviation (SD). These data were analyzed using the Kruskal-Wallis H test followed by the Nemenyi test. All other data are presented as the mean ± standard error of the mean (SEM). The transit time of the colon was analyzed using repeated-measures analysis of variance (ANOVA) with between-subject factors. Other data were compared using ANOVA followed by the least significant difference (LSD) test. The criterion for statistical significance was *P* < 0.05.

## Additional Information

**How to cite this article**: Fang, J.-f. *et al*. Electroacupuncture treatment partly promotes the recovery time of postoperative ileus by activating the vagus nerve but not regulating local inflammation. *Sci. Rep.* **7**, 39801; doi: 10.1038/srep39801 (2017).

**Publisher's note:** Springer Nature remains neutral with regard to jurisdictional claims in published maps and institutional affiliations.
